# A divide-and-conquer algorithm for large-scale *de novo* transcriptome assembly through combining small assemblies from existing algorithms

**DOI:** 10.1186/s12864-017-4270-9

**Published:** 2017-12-06

**Authors:** Sing-Hoi Sze, Jonathan J. Parrott, Aaron M. Tarone

**Affiliations:** 1 Department of Computer Science and Engineering, Texas A&M University, College Station, Mexico, 77843 TX USA; 2 Department of Biochemistry & Biophysics, Texas A&M University, College Station, Mexico, 77843 TX USA; 3 Department of Entomology, Texas A&M University, College Station, Mexico, 77843 TX USA

**Keywords:** Divide-and-conquer, RNA-Seq, *de novo* transcriptome assembly

## Abstract

**Background:**

While the continued development of high-throughput sequencing has facilitated studies of entire transcriptomes in non-model organisms, the incorporation of an increasing amount of RNA-Seq libraries has made *de novo* transcriptome assembly difficult. Although algorithms that can assemble a large amount of RNA-Seq data are available, they are generally very memory-intensive and can only be used to construct small assemblies.

**Results:**

We develop a divide-and-conquer strategy that allows these algorithms to be utilized, by subdividing a large RNA-Seq data set into small libraries. Each individual library is assembled independently by an existing algorithm, and a merging algorithm is developed to combine these assemblies by picking a subset of high quality transcripts to form a large transcriptome. When compared to existing algorithms that return a single assembly directly, this strategy achieves comparable or increased accuracy as memory-efficient algorithms that can be used to process a large amount of RNA-Seq data, and comparable or decreased accuracy as memory-intensive algorithms that can only be used to construct small assemblies.

**Conclusions:**

Our divide-and-conquer strategy allows memory-intensive *de novo* transcriptome assembly algorithms to be utilized to construct large assemblies.

## Background

While high-throughput sequencing has made it possible to perform studies of entire transcriptomes in non-model organisms, applying *de novo* transcriptome assembly algorithms has been increasingly difficult due to an increasing amount of RNA-Seq libraries that include many experimental conditions or developmental stages with replicated experiments.

**Fig. 1:**
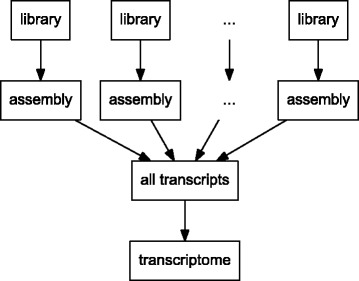
Illustration of the divide-and-conquer strategy. A large RNA-Seq data set is subdivided into small libraries. Each individual library is assembled independently, and a merging algorithm is employed to combine the small assemblies into a large transcriptome

Although transcriptome assembly algorithms such as SOAPdenovo-Trans [1] and Trans-ABySS [2] can be used to process a large amount of RNA-Seq data, algorithms such as Oases [3] and Trinity [4] that have higher accuracy are generally very memory-intensive, thus they can only be used to construct small assemblies. We develop a divide-and-conquer strategy that allows these algorithms to be utilized. A large RNA-Seq data set is subdivided into small libraries. Each individual library is assembled independently, and a merging algorithm is employed to combine the small assemblies into a large transcriptome (Fig. 1).

The merging algorithm picks a subset of high quality transcripts to form a transcriptome by preferring longer transcripts, which are more highly expressed and better assembled. A de Bruijn graph is constructed to extend some of these transcripts at the left end and at the right end when there are no ambiguities. To reduce redundancy, lower ranked transcripts with all their corresponding nodes in the de Bruijn graph covered by higher ranked transcripts are removed.

We validate our algorithm by performing *Schizosaccharomyces pombe*, *Drosophila melanogaster* and *Arabidopsis thaliana* transcriptome assemblies using publicly available RNA-Seq libraries. We demonstrate our algorithm by assembling a large set of 93 *Cochliomyia macellaria* RNA-Seq libraries that we have constructed, which is about 298 G in size.

## Methods

### De Bruijn graph construction

Given a set of reads and a parameter *k* that denotes the *k*-mer length, a de Bruijn graph is defined by taking each *k*-mer that appears in the reads as a vertex, and connecting two *k*-mers *s*_1_ *s*_2_⋯*s*_*k*_ and *s*_2_⋯*s*_*k*_ *s*_*k*+1_ by a directed edge if the (*k*−1)-suffix of the first *k*-mer is the same as the (*k*−1)-prefix of the second *k*-mer and the (*k*+1)-mer *s*_1_ *s*_2_⋯*s*_*k*_ *s*_*k*+1_ appears in the reads.

Since the de Bruijn graph implicitly represents an assembly of the reads, it is employed by short read assembly algorithms [5, 6]. To reduce noise, a *k*-mer coverage cutoff *c* is imposed to remove *k*-mers that appear less than *c* times. Each maximal non-branching linear path is collapsed into a single node, thus each node can contain a longer string that is formed from concatenating successive *k*-mers that overlap by *k*−1 letters between each adjacent pair. After each individual library is assembled independently using an existing algorithm, our algorithm constructs a de Bruijn graph from the set of all predicted transcripts. Note that the *k*-mer coverage cutoff *c* is only applied during individual library assemblies and not during the merging step.

To construct the de Bruijn graph, we follow the iterative one-letter extension strategy in [7] to identify all *k*-mers. Given a sorted array that contains all *n* *k* ^′^-mers in either the forward or the reverse complementary direction for *k* ^′^<*k*, an array of size 4*n* is created that contains four slots for each *k* ^′^-mer. For each (*k* ^′^+1)-mer, binary search is applied to locate its *k* ^′^-prefix within the array and one of the four slots that corresponds to its last nucleotide is updated. Slots with zero counts are removed to obtain all (*k* ^′^+1)-mers. Edges in the de Bruijn graph are constructed by locating the corresponding *k*-mers through binary search. Since this step is performed on the transcripts and not on the reads, it is not time consuming and the memory requirement has a multiplicative constant of four per *k*-mer.

### Picking high quality transcripts

Since each transcript corresponds to a path in the de Bruijn graph and there may be extra bases at the beginning node and the ending node of the path that are not included within the transcript, these bases form unambiguous extensions of the transcript and are added to the transcript. To reduce noise, we only retain a transcript if its length divided by the number of nodes in its path is above the average length cutoff *c*_1_, where *c*_1_ is a given parameter. To form the transcriptome from these extended transcripts, our algorithm picks a subset of high quality transcripts while preferring longer transcripts.

Since the longest transcripts are not always correct and may have translocations, and we have to make sure that redundant subsequences of a transcript are not included multiple times, we rank the transcripts in decreasing order of the number of nodes that form each transcript in the de Bruijn graph. Since transcripts that are formed from a larger number of nodes in the de Bruijn graph tend to be longer, this strategy has a preference towards longer transcripts while at the same time reduces the number of translocated transcripts.

To remove redundant transcripts, we consider long nodes in the de Bruijn graph that contain a string of length above the node length cutoff *c*_2_, where *c*_2_ is another given parameter. We only retain a lower ranked transcript when it contains a long node that is not covered by higher ranked transcripts. We group transcripts into a locus when they share at least one long node. This condition is applied transitively to collect all related transcripts so that each transcript in a locus shares at least one long node with another transcript in the same locus.

## Results and discussion

### Data sets

We applied our algorithm kCombine to perform transcriptome assemblies using publicly available RNA-Seq libraries from the sequence read archive [8], including one set of *Schizosaccharomyces pombe* libraries, two sets of *Drosophila melanogaster* libraries with one small set and one large set, and one set of *Arabidopsis thaliana* libraries (Table 1).

**Table 1:** Data sets used in the evaluation, with organism denoting the organism, type denoting whether the organism is model or non-model, libraries denoting the total number of libraries with the number in parentheses denoting the number of libraries after combining the biological replicates for independent assembly in our algorithm, size denoting the total number of bases in all the reads after quality trimming, and reference denoting the publication that describes the libraries

Organism	Type	Libraries	Size	Reference
*Schizosaccharomyces pombe*	Model	32	16.9 G	[4]
*Drosophila melanogaster*	Model	13	9.4 G	[18]
*Arabidopsis thaliana*	Model	5	16.1 G	[19]
*Drosophila melanogaster*	Model	245 (34)	158 G	[20]
*Cochliomyia macellaria*	Non-model	93 (31)	298 G	New data

We compare the performance of our algorithm that utilizes an existing algorithm to assemble each individual library independently to the same algorithm that returns a single assembly directly from all libraries, with each library corresponding to one sequencing run of a biological sample and all biological replicates combined into a single library for independent assembly in our algorithm. We trimmed each read by removing bases starting from the first position that has a quality score of less than 15. We applied Oases and Trinity to the small data sets, and SOAPdenovo-Trans and Trans-ABySS to the large data sets.

We fixed the *k*-mer length to 25 and varied the *k*-mer coverage cutoff *c* when applying each algorithm. We used the same value of *k* to construct the de Bruijn graph in our algorithm, and set the average length cutoff *c*_1_ to 25 and the node length cutoff *c*_2_ to 50. These parameters were determined by trying a few combinations and choosing the values that give satisfactory performance. Since the performance of each algorithm is highly dependent on the *k*-mer coverage cutoff *c* and different values are needed when applying an existing algorithm during the divide-and-conquer strategy as opposed to obtaining a single assembly directly, we report the results that give the most comparable performance.

To assess the extent of translocations in predicted transcripts, we applied GMAP [9] to map the predicted transcripts to the known genome. To investigate whether our algorithm may systematically remove certain types of RNA, we applied eXpress [10] to the reads in each data set with respect to all the predicted transcripts that are full length transcripts in each assembly to obtain FPKM expression estimates.

### Model organisms

Tables 2 and 3 show that kCombine generally had decreased performance when compared to obtaining single assemblies directly from Oases or Trinity. When compared to Oases, kCombine was able to obtain less translocated transcripts in *Schizosaccharomyces pombe* and comparable percentages of translocated transcripts in *Drosophila melanogaster*. When compared to Trinity, kCombine had decreased performance when the percentage of translocated transcripts is about the same, and kCombine had a higher percentage of translocated transcripts when the other performance is about the same. Tables 4 and 5 show that kCombine had improved performance when compared to SOAPdenovo-Trans and Trans-ABySS at the expense of having more translocated transcripts.

**Table 2:** Comparisons of *Schizosaccharomyces pombe* transcriptome assemblies of Oases, Trinity and kCombine with *k*=25 over different values of *k*-mer coverage cutoff *c*, with transcripts denoting the number of predicted transcripts, n50 denoting the N50 value of the length of predicted transcripts, blast denoting the number of hits from nucleotide BLAST search of predicted transcripts to different transcripts of the known transcriptome with *e*-value below 10^−100^, full denoting the number of predicted transcripts that are full length transcripts in which an entire coding region is included in a BLAST alignment, spec denoting the percentage of positions in the predicted transcripts that are included in a BLAST alignment, unique denoting the number of predicted transcripts that are uniquely mapped as reported by GMAP with the percentage in parentheses, transloc denoting the number of predicted transcripts that are translocated as reported by GMAP with the percentage in parentheses, and memory denoting the physical memory requirement as a power of 2 with Trinity using 32 CPU

*c*	Transcripts	n50	Blast	Full	Spec	Unique	Transloc	Memory
Oases								
10	8244	6520	6515	5623	91.9%	7268 (88.2%)	1084 (13.1%)	128 GB
20	6509	5412	6458	4597	93.1%	5959 (91.6%)	672 (10.3%)	128 GB
kCombine(Oases)								
5	8585	2705	6227	5223	94.8%	8019 (93.4%)	646 (7.5%)	8 GB
10	6603	2620	5767	4443	95.2%	6173 (93.5%)	472 (7.1%)	8 GB
Trinity								
5	8568	3368	6490	5279	93.7%	8228 (96.0%)	150 (1.8%)	512 GB
10	7313	2604	6415	4271	95.1%	7064 (96.6%)	156 (2.1%)	512 GB
kCombine(Trinity)								
3	6862	2319	6152	3858	96.5%	6628 (96.6%)	262 (3.8%)	8 GB
5	6618	1938	5898	3182	97.1%	6415 (96.9%)	166 (2.5%)	8 GB

**Table 3:** Comparisons of small *Drosophila melanogaster* transcriptome assemblies of Oases, Trinity and kCombine with *k*=25 over different values of *k*-mer coverage cutoff *c*

*c*	Transcripts	n50	Blast	Full	Spec	Unique	Transloc	Memory
Oases								
10	41867	1743	24187	8444	91.6%	36080 (86.2%)	7026 (16.8%)	128 GB
20	38377	1164	21606	4922	88.1%	34926 (91.0%)	3830 (10.0%)	128 GB
kCombine(Oases)								
5	52282	1144	23573	7152	89.9%	44118 (84.4%)	9938 (19.0%)	8 GB
10	36813	939	20389	4430	88.3%	32591 (88.5%)	5272 (14.3%)	8 GB
Trinity								
5	35190	1313	24434	6177	95.7%	32481 (92.3%)	2664 (7.6%)	512 GB
10	29237	970	22388	3986	96.9%	27883 (95.4%)	978 (3.3%)	512 GB
kCombine(Trinity)								
3	34150	884	22746	4097	96.0%	31540 (92.4%)	2558 (7.5%)	8 GB
5	26535	753	20525	2932	96.4%	24994 (94.2%)	1214 (4.6%)	8 GB

Notations are the same as in Table 2

**Table 4:** Comparisons of *Arabidopsis thaliana* transcriptome assemblies of SOAPdenovo-Trans, Trans-ABySS and kCombine with *k*=25 over different values of *k*-mer coverage cutoff *c*

*c*	Transcripts	n50	Blast	Full	Spec	Unique	Transloc	Memory
SOAPdenovo-Trans								
20	88808	841	27325	1583	78.7%	76542 (86.2%)	44 (0.0%)	4 GB
50	59638	887	24554	1302	82.9%	51402 (86.2%)	38 (0.1%)	4 GB
kCombine(SOAPdenovo-Trans)								
3	88611	1103	29057	2666	81.7%	81576 (92.1%)	1324 (1.5%)	4 GB
5	70854	1125	28020	2354	84.1%	65574 (92.5%)	972 (1.4%)	4 GB
Trans-ABySS								
20	124125	830	28414	4372	88.0%	119120 (96.0%)	1574 (1.3%)	2 GB
50	62093	1006	25533	3109	91.9%	59420 (95.7%)	1010 (1.6%)	2 GB
kCombine(Trans-ABySS)								
3	90109	1147	30333	3261	82.2%	83037 (92.2%)	4242 (4.7%)	4 GB
5	59411	1315	29266	3303	88.0%	54903 (92.4%)	3464 (5.8%)	4 GB

Notations are the same as in Table 2

**Table 5:** Comparisons of large *Drosophila melanogaster* transcriptome assemblies of SOAPdenovo-Trans, Trans-ABySS and kCombine with *k*=25 over different values of *k*-mer coverage cutoff *c*

*c*	Transcripts	n50	Blast	Full	Spec	Unique	Transloc	Memory
SOAPdenovo-Trans								
20	92193	1272	27258	6926	72.1%	81426 (88.3%)	186 (0.2%)	16 GB
50	65273	1422	26671	6832	81.8%	58101 (89.0%)	90 (0.1%)	16 GB
kCombine(SOAPdenovo-Trans)								
3	84307	1944	27391	12269	79.6%	77649 (92.1%)	1222 (1.4%)	4 GB
5	66098	2037	27153	11710	84.0%	61227 (92.6%)	976 (1.5%)	4 GB
Trans-ABySS								
20	99492	1581	27631	11103	75.7%	90854 (91.3%)	5118 (5.1%)	8 GB
50	62731	1980	27262	12151	86.0%	57508 (91.7%)	2786 (4.4%)	8 GB
kCombine(Trans-ABySS)								
3	119772	2628	27612	22113	79.3%	99021 (82.7%)	22638 (18.9%)	4 GB
5	80966	3009	27612	23110	84.5%	64161 (79.2%)	22012 (27.2%)	4 GB

Notations are the same as in Table 2

In terms of memory requirement, while Oases and Trinity required much more memory than kCombine, the memory requirement of SOAPdenovo-Trans and Trans-ABySS was comparable to kCombine. More memory was needed during the merging step by kCombine than the independent assembly of each individual library by each algorithm.

**Fig. 2:**
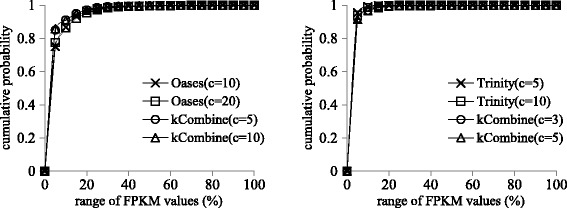
Comparisons of the cumulative distribution of the FPKM expression estimates of predicted transcripts that are full length transcripts in *Schizosaccharomyces pombe* transcriptome assemblies of Oases, Trinity and their respective applications of kCombine, with *k*=25 over different values of *k*-mer coverage cutoff *c* and the range of FPKM values in each assembly divided into 20 intervals of equal width

**Fig. 3:**
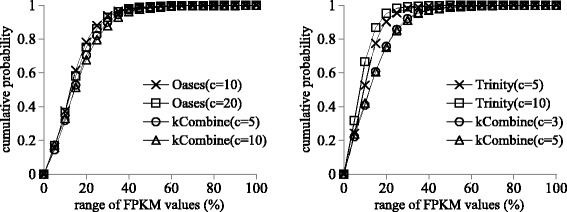
Comparisons of the cumulative distribution of the FPKM expression estimates of predicted transcripts that are full length transcripts in small *Drosophila melanogaster* transcriptome assemblies of Oases, Trinity and their respective applications of kCombine, with *k*=25 over different values of *k*-mer coverage cutoff *c* and the range of FPKM values in each assembly divided into 20 intervals of equal width

**Fig. 4:**
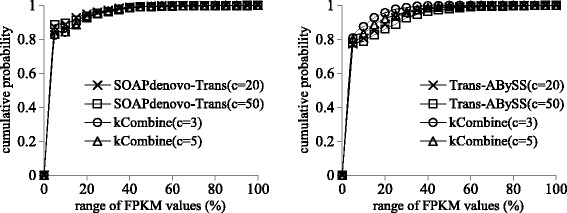
Comparisons of the cumulative distribution of the FPKM expression estimates of predicted transcripts that are full length transcripts in *Arabidopsis thaliana* transcriptome assemblies of SOAPdenovo-Trans, Trans-ABySS and their respective applications of kCombine, with *k*=25 over different values of *k*-mer coverage cutoff *c* and the range of FPKM values in each assembly divided into 20 intervals of equal width

**Fig. 5:**
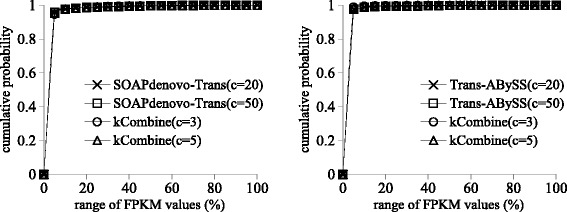
Comparisons of the cumulative distribution of the FPKM expression estimates of predicted transcripts that are full length transcripts in large *Drosophila melanogaster* transcriptome assemblies of SOAPdenovo-Trans, Trans-ABySS and their respective applications of kCombine, with *k*=25 over different values of *k*-mer coverage cutoff *c* and the range of FPKM values in each assembly divided into 20 intervals of equal width

Figures 2 and 3 show that kCombine recovered comparable proportion of full length transcripts with low expression levels as Oases and Trinity, with slightly higher proportion than Oases in *Schizosaccharomyces pombe* and lower proportion than Trinity in *Drosophila melanogaster*. Figures 4 and 5 show that kCombine recovered comparable proportion of full length transcripts with low expression levels as SOAPdenovo-Trans and Trans-ABySS, with slightly lower proportion than SOAPdenovo-Trans and higher proportion than Trans-ABySS in *Arabidopsis thaliana*.

### Non-model organism

We applied our algorithm to assemble the transcriptome of the blow fly *Cochliomyia macellaria* from a set of RNA-Seq libraries that we have constructed (Table 1), in which the full transcriptome was not available before. The blow fly *Cochliomyia macellaria* is a primary colonizer of human and animal remains, and is important in nutrient cycling [11, 12] and forensic investigations of deaths [13, 14]. As an agent of myiasis [15, 16] and as a filth feeding fly, this species can be a veterinary and medical pest by causing direct damage to hosts and by spreading pathogenic bacteria [17]. Genomic tools for this blow fly can be expected to improve the benefits of *Cochliomyia macellaria* biology and to ameliorate the negative aspect.

Three biological replicates were generated for each combination of one of four temperatures (20 °C, 25 °C, 30 °C, and fluctuated) and one of seven developmental stages (feeding instar, early post feeding, late post feeding, early pupae, early middle pupae, late middle pupae, and late pupae). We also include additional libraries that were selected for fast and slow development and a control sample.

Since our goal is to obtain a transcriptome that is as accurate and complete as possible, we applied kCombine based on Oases and Trinity due to their generally higher accuracy in terms of specificity. Table 6 shows that the assemblies were of high quality. In both cases of kCombine based on Oases and Trinity, the assembly based on the middle value of the *k*-mer coverage cutoff *c* provides a balanced result between sensitivity and correctness. In terms of memory requirement, Oases and Trinity required a large amount of memory during the independent assembly of each individual library. Since the total size of libraries is very large, the assembly of each individual library is difficult even when the data set is divided into 31 libraries after combining the biological replicates. The merging step by kCombine required comparably little memory.

**Table 6:** Transcriptome assemblies of kCombine based on Oases and Trinity in *Cochliomyia macellaria* with *k*=25 over different values of *k*-mer coverage cutoff *c*, with locus denoting the number of predicted locus, transcripts denoting the number of predicted transcripts, n50 denoting the N50 value of the length of predicted transcripts, blastx denoting the number of hits from translated BLAST search of predicted transcripts to different transcripts of the known *Drosophila melanogaster* transcriptome with *e*-value below 10^−20^, and memory denoting the physical memory requirement as a power of 2, with the value to the left of “+” indicating the memory requirement of the independent assembly of each individual library by Oases and Trinity, and the value to the right of “+” indicating the memory requirement of the merging step by kCombine

*c*	Locus	Transcripts	n50	Blastx	Memory
kCombine(Oases)					
10	60477	140681	2502	20385	256 GB + 4 GB
20	42334	101421	2349	20579	256 GB + 4 GB
50	30858	67217	1924	20101	256 GB + 4 GB
kCombine(Trinity)					
5	46342	153790	1961	22996	512 GB + 16 GB
10	34614	111379	1934	22874	512 GB + 16 GB
20	28756	85093	1567	22595	512 GB + 16 GB

## Conclusions

We have developed a divide-and-conquer strategy that allows memory-intensive *de novo* transcriptome assembly algorithms to be utilized to construct large assemblies. After the individual libraries are assembled independently, the merging algorithm consumes little computational time and memory. In all our tests, the independent assembly of each individual library can be completed in a few days when performed in parallel on a computing cluster. The merging step then takes up to a few days for the largest data sets.

The choice of which algorithm to use depends on the goal of the assembly. While the memory requirement can still be high even after applying the divide-and-conquer strategy on memory-intensive algorithms for very large data sets, they are generally more accurate, with Oases returning more and longer transcripts and Trinity returning more transcripts with low expression levels and with less translocations. Among the memory-efficient algorithms, SOAPdenovo-Trans returns transcripts with less translocations while Trans-ABySS returns more and longer transcripts with higher specificity.

Since there is a decrease in performance in the divide-and-conquer strategy as the number of libraries increases, it is better to subdivide into smaller number of libraries as long as there are enough computational resources to assemble them independently. To optimize the performance, different values of the *k*-mer coverage cutoff *c* can be used on libraries of different sizes.
